# Meibum Lipid Composition in Asians with Dry Eye Disease

**DOI:** 10.1371/journal.pone.0024339

**Published:** 2011-10-17

**Authors:** Sin Man Lam, Louis Tong, Siew Sian Yong, Bowen Li, Shyam S. Chaurasia, Guanghou Shui, Markus R. Wenk

**Affiliations:** 1 Department of Biological Sciences, National University of Singapore, Singapore, Singapore; 2 Singapore Eye Research Institute, Singapore, Singapore; 3 Singapore National Eye Centre, Singapore, Singapore; 4 Duke-NUS Graduate Medical School, Singapore, Singapore; 5 Yong Loo Lin School of Medicine, Department of Biochemistry, National University of Singapore, Singapore, Singapore; 6 Life Sciences Institute, National University of Singapore, Singapore, Singapore; Alcon Research, Ltd., United States of America

## Abstract

**Background:**

Previous lipidomic analyses of the human meibum had largely focused on individuals from non-Asian populations, despite the higher prevalence of dysfunctional tear syndrome (DTS) observed across Asia. Information pertaining to the alterations in lipid profiles in relation to DTS onset and progression is also lacking and warrants comprehensive experimental analysis.

**Methodologies/Principal Findings:**

We examined the meibum lipidome of 27 DTS patients and 10 control subjects for a total of 256 lipid species from 12 major lipid classes, including cholesteryl ester (CE), wax ester (WE), triacylglyceride (TAG), (O-acyl)-ω-hydroxy fatty acid (OAHFA), glycerophospholipids (phosphatidylcholine, PC; phosphatidylethanolamine, PE; phosphatidylinositol, PI; phosphatidylglycerol, PG) and sphingolipids (sphingomyelin, SM; ceramide, Cer; glucosylceramide, GluCer; dihexosylceramide, DihexCer). Neutral lipids were analysed using high-performance liquid-chromatography coupled with mass spectrometry (HPLC/MS) and tandem mass spectrometry (MS/MS) was used for the qualitative and quantitative analysis of polar lipid species. DTS patients were classified into three severity groups (*i.e.* mild, moderate and severe) based on the ocular surface disease index (OSDI). A significantly lower level of TAG (p<0.05) was observed in patients under the moderate category compared to the mild category. Notably, a number of OAHFA species displayed consistently decreasing levels that correlate with increasing disease severity. An attempt was also made to investigate the changes in meibum lipid profiles of DTS patients compared to normal individuals classified based on OSDI score. Several unsaturated TAG and PC species were found at significantly higher levels (p<0.05) in patients than controls.

**Conclusion:**

The current study presents, for the first time, a comprehensive lipidome of meibum from individuals of an Asian ethnicity, which can potentially offer new insights into the higher prevalence of DTS observed amongst Asian populations. This study also represents an attempt towards identification of lipid species in meibum which could serve as marker for DTS.

## Introduction

Dysfunctional tear syndrome (DTS), more commonly known as dry eye disease, is a multifactorial disease of the tears and ocular surface which is primarily caused by tear deficiency or excessive evaporation. It is often accompanied by an increase in tear film osmolarity and ocular surface inflammation that can be potentially damaging to the ocular surface. [1]. DTS also represents a highly prevalent disease which can affect up to approximately a third of the population worldwide [1]–[3], depending on the criteria and definition used in the various studies conducted across the continents. Recent studies conducted in China and Japan had, however, yielded much higher prevalence than the average value reported globally [4], [5], implying that the Asian populations might have a greater predisposition to the disease.

The structural integrity and stability of the tear film is intricately linked to the development and progression of DTS. The tear film constitutes the interface between the ocular surface and its immediate external environment, and thus plays a critical role in protecting the eye from a myriad of environmental stresses. The tear film is composed of three basic layers – the carbohydrate-rich glycocalyx layer where the apical microvilli of the superficial corneal epithelial cells resides; an intermediate aqueous layer largely contributed by aqueous secretion from the lacrimal glands that constitutes the bulk of the tear film; as well as the most superficial lipid layer in immediate contact with the external environment [6]. The tear film lipid layer (TFLL) comprises the outer, superficial sublayer consisting predominantly of non-polar lipids and an inner, amphiphilic layer which serves as a boundary between the non-polar lipid sublayer and the polar aqueous layer underneath. The amphiphilic lipid sublayer facilitates the interaction between the two layers to ensure the even spreading of the non-polar lipid sublayer during each blink of the eye [6]–[9]. The TFLL plays a major role in the pathogenesis of the evaporative dry eye due to its chief function in retarding evaporation from the underlying aqueous layer [6]. Therefore, an analytical examination of the compositional changes of the TFLL is of paramount importance to the understanding of DTS pathogenesis and also in facilitating the development of better diagnostic tools and treatment strategies.

Secretion from the meibomian glands, or the meibum, constitutes the cardinal source of lipids for the TFLL in humans [6], [8]. Several studies in the past decades had shown that the human meibum is made up of a highly complex mixture of lipids from various classes [7], [8], [10]–[14], which had posed a considerable amount of challenges in elucidation of its composition. The limiting quantity of sample (typically in the range of a few milligrams) that can be obtained from donors further exacerbated the progress in the compositional evaluation of human meibum.

The recent decades have witnessed a rapid increase in the number of publications pertaining to meibomian and tear lipids [15], which is partly attributed to the technological advancements in the area of mass spectrometry [7], [8], [15]–[25]. While these studies had contributed to constructing a more comprehensive view of human meibum composition, most of these studies were, however, circumscribed by a relatively small sample size, or a lack of clinical and demographic information pertaining to the individuals from which lipid samples were collected [19]–[21], [23]. Moreover, few of these studies had analyzed and compared the meibum lipid compositions between normal volunteers and individuals suffering from DTS, except for a study reported by Joffre and coworkers [7] in which a comparison on the fatty acid composition in the meibum between normal individuals and those suffering from meibomian gland dysfunction was made. Furthermore, all the aforementioned studies had emphasized on the analyses of meibum lipids collected from non-Asian populations, and very limited data currently exist on the potential effect of race or ethnicity on dry eye prevalence. Data from the Women's Health Study had, however, suggested that the prevalence of severe symptoms and/or clinical diagnosis of dry eye might be greater in Hispanic and Asian as compared to Caucasian individuals [26].

It must be noted that the complexities in the manifestations of DTS *per se* and its highly multifactorial nature have presented a considerable amount of challenges in the clinical classification and diagnosis of the disease. The difficulty in DTS diagnosis has, to a large extent, hindered the development of new pharmacological therapies due to a lack of objective tests for response outcomes [27], [28]. This may also be an explanation for the relative scarcity of case-control studies (see above). Nevertheless, one must recognise that it is this very lack of definite clinical markers that necessitates the search for novel biomarker(s) that can provide greater sensitivity and specificity to facilitate disease diagnosis and treatment monitoring. Recently, Sullivan and coworkers had evaluated the different clinical indicators commonly used to categorize varying degrees of DTS, and a comprehensive scheme encompassing the myriad of clinical markers was consolidated. The group generated a composite index of disease severity, which has standardized clinical definitions of DTS disease severity by providing the corresponding threshold values for each clinical marker that distinctly define each severity level [29].

In the current study, dry eye patients were classified predominantly based on a scale of symptom severity. The Ocular Surface Disease Index (OSDI) symptom questionnaire was used for symptom assessment [30]. Compared to other standard clinical tests, diagnosis of dry eye based on symptom severity might possibly confer better indication of early-stage ocular surface distress. Moreover, symptomatic evaluation of the disease nonetheless represents the best way to monitor disease progression and treatment effectiveness as far as the quality of life of any given patient is concerned [31]. While there is no general consensus over which dry eye questionnaire is most comprehensive in terms of symptom coverage, or which symptom is best correlated to disease pathogenesis and progression, symptomatic classification of patients can represent a novel way for preliminary analysis of the possible lipid biomarker(s) that may correlate with the observed symptom, and which may therefore play an important role in disease pathogenesis. In the current study, the OSDI cut-offs reported by Sullivan et al. were used as a reference to stratify patients into different groups with different severity levels, and also as a crucial criterion for selection of DTS patients and normal controls in a more stringent manner apart from other commonly used clinical indicators such as the tear breakup time (TBUT) [29].

**Figure 1:**
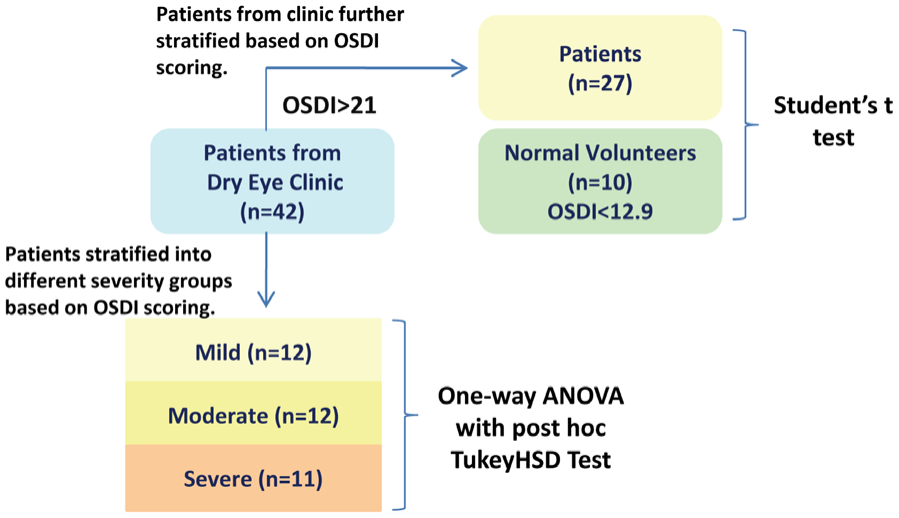
Flow-chart illustrating the different schemes of statistical analysis used in the current study design.

The present study describes the first comprehensive report on the lipid profiles of meibum obtained from individuals of an Asian origin, mainly Chinese in ethnicity. We present a comprehensive lipidome of the human meibum comprising a total of 256 lipid species from 12 major lipid classes investigated. Comparisons were also made on the quantitative and qualitative differences in lipid profiles of meibum amongst patients suffering from varying degrees of DTS, as well as between normal volunteers and patients (Figure 1), in an attempt to elucidate potential lipid marker(s) that can possibly confer new insights into the diagnosis, monitoring as well as pathogenesis of DTS.

## Results

### Lipidome of human meibum

**Figure 2:**
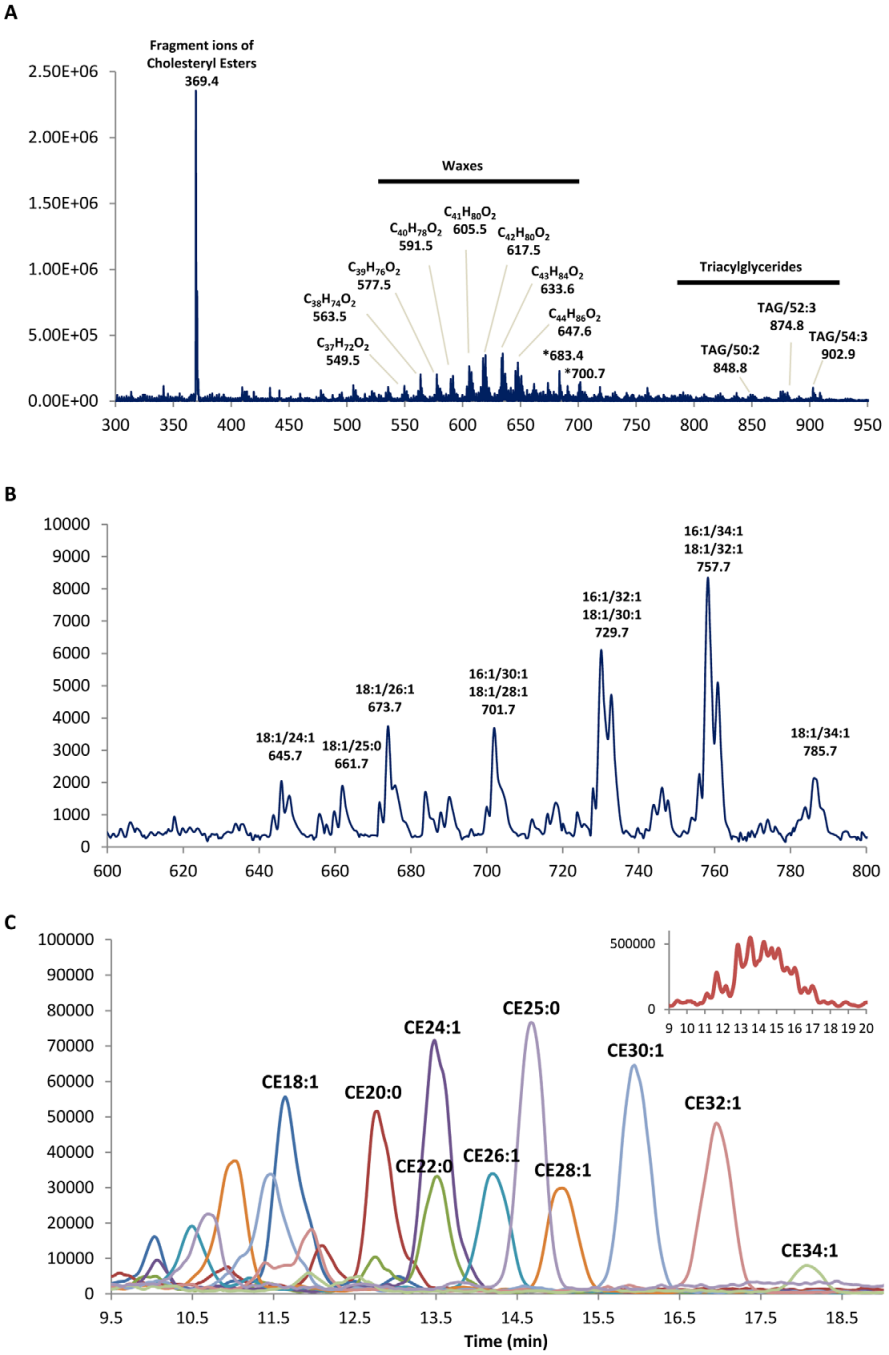
Preliminary mass spectrometric data of the human meibum. Single-stage MS profile of human meibum in the (A) positive ion mode and (B) negative ion mode. (C) Representative EIC of selected CE species eluted in the order of increasing hydrophobicity. Insert: EIC (m/z = 369.4) of all cholesteryl ester species present. *Denotes background peak.

Single-stage mass spectrometric profile in the positive ion mode revealed that the non-polar lipid classes of cholesteryl ester (CE), wax ester (WE) and triacylglyceride (TAG) comprise the bulk of human meibum lipids (Figure 2A), constituting approximately 90% of the total lipids present (Table 1). On the other hand, the class of (O-acyl)-ω-hydroxy fatty acid (OAHFA) constitutes the majority of the polar lipid fraction detected in the negative ion mode (Figure 2B), making up approximately 3.5% of the total meibum lipids, followed by various subclasses of phospholipids (phosphatidylcholine, PC; phosphatidylethanolamine, PE; phosphatidylinositol, PI; phosphatidylglycerol, PG) and sphingolipids (sphingomyeloin, SM; ceramide, Cer; glucosylceramide, GluCer; dihexosylceramide, DihexCer) that collectively comprise less than 1% of the total meibum lipids (Table 1).

**Table 1:** Historical overview on the distribution of major lipid classes in human meibum.

Lipid(% of total lipids)	Tiffany(1978)^a^ ^[11]^	Nicolaides(1981)^[12]^	McCulley(1997)^[13]^	Mathers (1998)^[14]^	Butovich(2007–2009)^[8,20]^	Chen(2010)^[24]^	Current(2011)
**SE**	8–34	29.50	16	39.4	30	13	66.83
**WE**	13–23	34.96	68	51.1	-	28	25.21*
**TAG**	11–43	4.00	6	3.1	-	0.05	4.03
**DE**	-	8.37	-	2.3	-	-	-
**FFA**	0–24	2.14	1	2.8	-	3	-
**PL** ‡	0–5	16.04^b^(polar lipids)	4	-	<0.015if any	-	0.37
**SL** †	-	16.04^b^(polar lipids)	1.5^c^	-	-	-	0.10
**OAHFA**	-	-	-	-	-	-	3.46

SE, sterol ester; WE, wax ester; TAG, triacylglycerides; DE, diesters; FFA, free fatty acid; PL, phospholipid; SL, sphingolipid; OAHFA, (O-acyl)-ω-hydroxy-fatty acid.

‡ PL refers to sum total of PC, phophatidylcholine; PE, phosphatidylethanolamine; PI, phosphatidylinositol; PG, phosphatidylglycerol.

† SL refers to sum total of SM, sphingomyelin; CER, ceramides; GluCer, glucosylceramide; DihexCer, dihexosylceramide. Values presented for the current study represent percentages of individual lipid classes in the meibum from normal volunteers.

* The quantity of WE is subjected to the use of palmityl palmitate as an internal standard.

a Analyses were conducted on pooled samples.

b Estimate was given for total polar lipids excluding free fatty acids.

c Estimate referred specifically to cerebroside content.

### Distribution of non-polar lipids

The nonpolar lipid fraction of the meibum was largely represented by CE, WE and TAG (Table 1). CE represents a major component of the non-polar lipid fraction and comprised approximately 65% of the entire lipid pool in current analysis (Table 1). In the reverse-phase, individual species of CE was separated on the basis of increasing hydrophobicity due to increasing lengths of the aliphatic fatty acid chains (Figure 2C). At least 20 different species of CE were observed, with essentially long chain and very long chain fatty acid residues (LCFA and VLCFA) ranging from C16 to C32. Saturated and monounsaturated fatty acids were the most prominent compounds of the CE pool. The seven most abundant compounds of the CE family were CE18:1, CE20:0, CE24:1, CE24:0, CE25:0, CE26:0 and CE30:1 (Figure S1A). WE constituted around 25% of the total lipid pool in the meibum (Table 1), with total carbon number ranging from 35 to 48. Saturated and monounsaturated fatty alcohol moieties also predominated this class of lipids, with the four most abundant species represented by C_42_H_80_O_2_, C_42_H_82_O_2_, C_44_H_84_O_2_ and C_44_H_86_O_2_ (Figure S1B). Up to 40 different TAG species were detected, with triolein (TAG/54:3) being the most abundant species; other abundant species include TAG/49:2, TAG/50:2, TAG/52:3, TAG/52:2 and TAG/54:2 (Figure S1C). It was estimated that TAG comprised approximately 4% of the lipids detected in human meibum (Table 1). Notably, a number of highly-unsaturated TAG species (n = 4–9) were also detected in the current study, and their identities had been further confirmed by subsequent accurate mass analysis using the LTQ Orbitrap mass spectrometer (data not shown).

### Distribution of polar lipids

The class of OAHFA constitutes the bulk of the polar lipid fraction and consists of 28 distinct species, with C18:1/32:1, C18:1/30:1, C16:1/32:1 as the three species found in highest abundance (Figure S2). A number of isobaric species were found and their identities were confirmed using precursor ion scans in the negative ion mode (Figure 2B) as well as fatty acyl-based MRM approach (Table 2). To our knowledge, this is the first attempt at the quantification of OAHFA species using HPLC-MRM approach, which can provide more specific identification and therefore more accurate quantification of individual OAHFA species compared to shotgun lipidomic precursor ion scan analysis [24]. For instance, the OAHFA compositions of 46∶2, 48∶3, 48∶2, 49∶2, 50∶3, 50∶2 and 52∶3 were each composed of more than one distinct species. In concordance with previous studies [23], [24], oleic acid (C18:1) represents the predominant fatty acid found in this class of lipids, followed by C16:1, C18:2 and a small amount of C18:0 (Figure S2). PC represents the predominant class of phospholipids found in the human meibum, constituting approximately 0.2% of the total lipids present in the meibum of normal controls (Table 3). More than 40 different PC species were detected, with PC34:2, PC34:1 and PC36:4e/36:3p as the three most abundant species (Figure S3A). Up to 10 species of lysophosphatidylcholines (LPCs) that had not been previously reported were also detected, with LPC18:0e and LPC18:2 being present in appreciably greater quantities than other LPC as well as PC species (Figure S3A). For the first time, comprehensive lipid profiles for the classes of PE and PI were reported. Notably, lysophospholipids were present in greater abundance than the majority of other species within each class of phospholipids analyzed (Figure S3). For instance, LPE18:0p/18:1e and LPE18:0 represent the two most abundant lipid species within the class of PE (Figure S3B); while LPI18:0 and LPG18:0 were found in highest quantity within the lipid classes of PI and PG respectively (Figure S3C–D). A number of sphingolipids from the classes of SM, Cer, GluCer and DihexCer were also detected, making up approximately 0.10% of the total meibum lipids (Table 1, Figure S4).

**Table 2:** Summary of multiple reaction monitoring (MRM) transitions for individual OAHFA species.

Lipid Species	MRM transition	Lipid Species	MRM transition	Lipid Species	MRM transition
18∶1/24∶1	645.7/281.2	18∶2/30∶1	727.7/279.2	18∶1/32∶2	755.7/281.2
18∶1/24∶0	647.7/281.2	18∶1/30∶2	727.7/281.2	16∶1/34∶2	755.8/253.2
18∶1/25∶0	661.7/281.2	16∶1/32∶1	729.7/253.2	16∶1/34∶1	757.7/253.2
18∶1/26∶1	673.7/281.2	18∶1/30∶1	729.7/281.2	18∶1/32∶1	757.7/281.2
18∶1/26∶0	675.7/281.2	18∶1/31∶1	743.7/281.2	18∶2/33∶1	769.8/279.2
18∶0/27∶2	687.7/283.2	16∶1/33∶1	743.8/253.2	18∶1/33∶1	771.8/281.2
16∶1/30∶1	701.7/253.2	18∶1/31∶0	745.7/281.2	18∶2/34∶1	783.7/279.2
18∶1/28∶1	701.7/281.2	18∶2/32∶2	753.7/279.2	18∶1/34∶2	783.8/281.2
18∶1/28∶0	703.7/281.2	18∶2/32∶1	755.7/279.2	18∶1/34∶1	785.7/281.2
16∶1/32∶2	727.7/253.2				

**Table 3:** Comparison on the distribution of major lipid classes in the human meibum between normal subjects (n = 10) and DTS patients (n = 27).

	Percent Total Lipids (%)	
LIPID CLASS	Normal	Patient
Cholesteryl esters (CE)	66.834±3.413	67.755±1.924
Wax esters (WE)	25.214±2.564	23.877±1.733
Triacylglycerides (TAG)	4.031±0.519	4.549±0.314
Phosphatidylcholines (PC)	0.190±0.050	0.612±0.238
Phosphatidylethanolamines (PE)	0.154±0.044	0.136±0.017
Phosphatidylglycerols (PG)	0.002±0.001	0.002±0.000
Phosphatidylinositols (PI)	0.023±0.005	0.025±0.003
Sphingomyelins (SM)	0.032±0.005	0.061±0.014
Ceramides (CER)	0.031±0.005	0.040±0.008
Glucosylceramides (GluCer)	0.020±0.006	0.024±0.002
Dihexosylceramides (DihexCer)	0.013±0.004	0.011±0.001
O-acyl-ω-hydroxy-fatty acids (OAHFA)	3.458±0.485	2.909±0.266

### Comparison of meibum lipid profiles amongst patients of different severity levels

**Figure 3:**
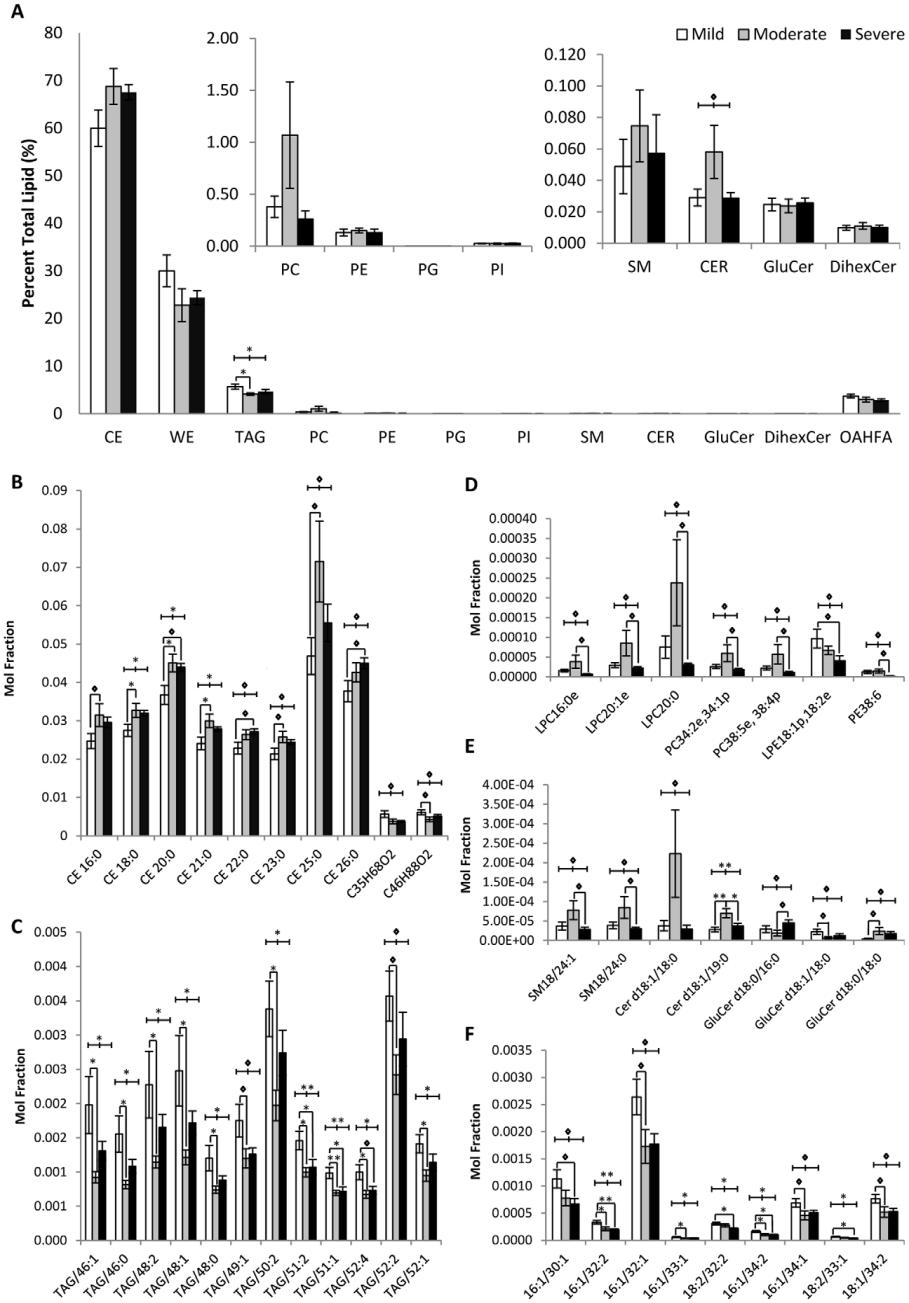
Quantitative comparison of lipid profiles between mild (n = 12), moderate (n = 12) and severe (n = 11) categories of DTS patients. (A) Comparison between total lipid classes. Insert, phospholipids (PC, PE, PG, PI) and sphingolipids (SM, Cer, GluCer, DihexCer). (B–F) Lipid species that were significantly different between the three categories. (B) CE and WE, cholesteryl esters and wax esters; (C) TAG, triacylglycerides; (D) Phospholipids; (E) Sphingolipids; (F) OAHFA, (O-acyl)-ω-hydroxy-fatty acids. ◊ p<0.10, * p<0.05, ** p<0.01.

There were no major differences in the overall distribution of lipids between the different categories of patients (Figure 3A). Similar to the general trend observed in meibum from normal controls, the non-polar lipid classes of CE, WE and TAG constitute the bulk of the meibum, and species of OAHFA form the majority of the polar lipid fraction. Interestingly, the total fraction of TAG was significantly different between patients for the three severity levels (p<0.05), with the total TAG fraction being significantly higher (p<0.05) in the mild category compared to the moderate category of patients. The level of total TAG increased slightly in the severe category compared to the moderate category but was not statistically significant.

Individual species of non-polar lipids that displayed statistically significant (p<0.05) and marginally significant (p<0.10) changes in quantities were shown in Figure 3B–C. It is noteworthy that most of the differences in individual non-polar lipid species were observed between patients from the mild and moderate categories. The levels of a number of CE species, including CE18:0, CE20:0 and CE21:0, were found in significantly higher levels (p<0.05) in the moderate than the mild category of patients (Figure 3B). Individual TAG species, on the other hand, displayed the opposite trend (Figure 3C). Consistent with the general trend observed for total TAG level, the levels of several TAG species, including TAG/46:1, TAG/46:0, TAG/48:2, TAG/48:1, TAG/48:0, TAG/50:2, TAG/51:2, TAG/51:1, TAG/52:4 and TAG/52:1, were significantly lower (p<0.05) in the moderate than the mild category of patients. The levels of majority of these TAG species increased slightly in the severe group compared to the moderate group, but were not of statistical significance.

Individual species of polar lipid species that showed statistically significant (p<0.05) and marginally significant (p<0.10) changes in quantities were shown in Figure 3D–F. In contrast to the changes observed in non-polar lipid species, most of the appreciable changes in phospholipid and sphingolipid species were found between the moderate and severe categories (Figure 3D–E). A number of LysoPCs, and other PC species containing double-bond(s) inherent in their structures *i.e.* ether/plasmalogen PCs or unsaturated PCs, were found in lower level in the severe compared to the moderate category of patients with marginal significance (p<0.10). Interestingly, the level of Cer d18:1/19:0 was significantly different (p<0.01) between the three categories of patients (Figure 3E); with a significantly higher level (p<0.05) observed in the moderate group than the mild group, and its level dropped significantly (p<0.05) in the severe group.

A number of OAHFA species, including 16∶1/32∶2, 16∶1/33∶1, 18∶2/32∶2 and 16∶1/34∶2 were observed to be significantly different (p<0.05) between the three categories of patients (Figure 3F). Notably, all these OAHFA species displayed a consistently decreasing trend as the severity level increases, although the levels between moderate and severe categories of patients were not statistically significant. Remarkably, the levels of 16∶1/32∶2 and 16∶1/34∶2 were significantly higher (p<0.05) in the mild category of patients compared to both the moderate and severe categories, respectively.

 There was no difference in the mean tear break up time (TBUT) of the patients in each level of severity by ANOVA (p>0.05) (Table S3). Similarly, there was no difference in the viscosity of the meibum or regularity of the gland orifices by clinical examination between these groups. There was no appreciable difference in the extent of eyelid notching or telangiectasia in these patients (data not shown).

### Comparison of meibum lipid profiles between DTS patients and normal controls

**Figure 4:**
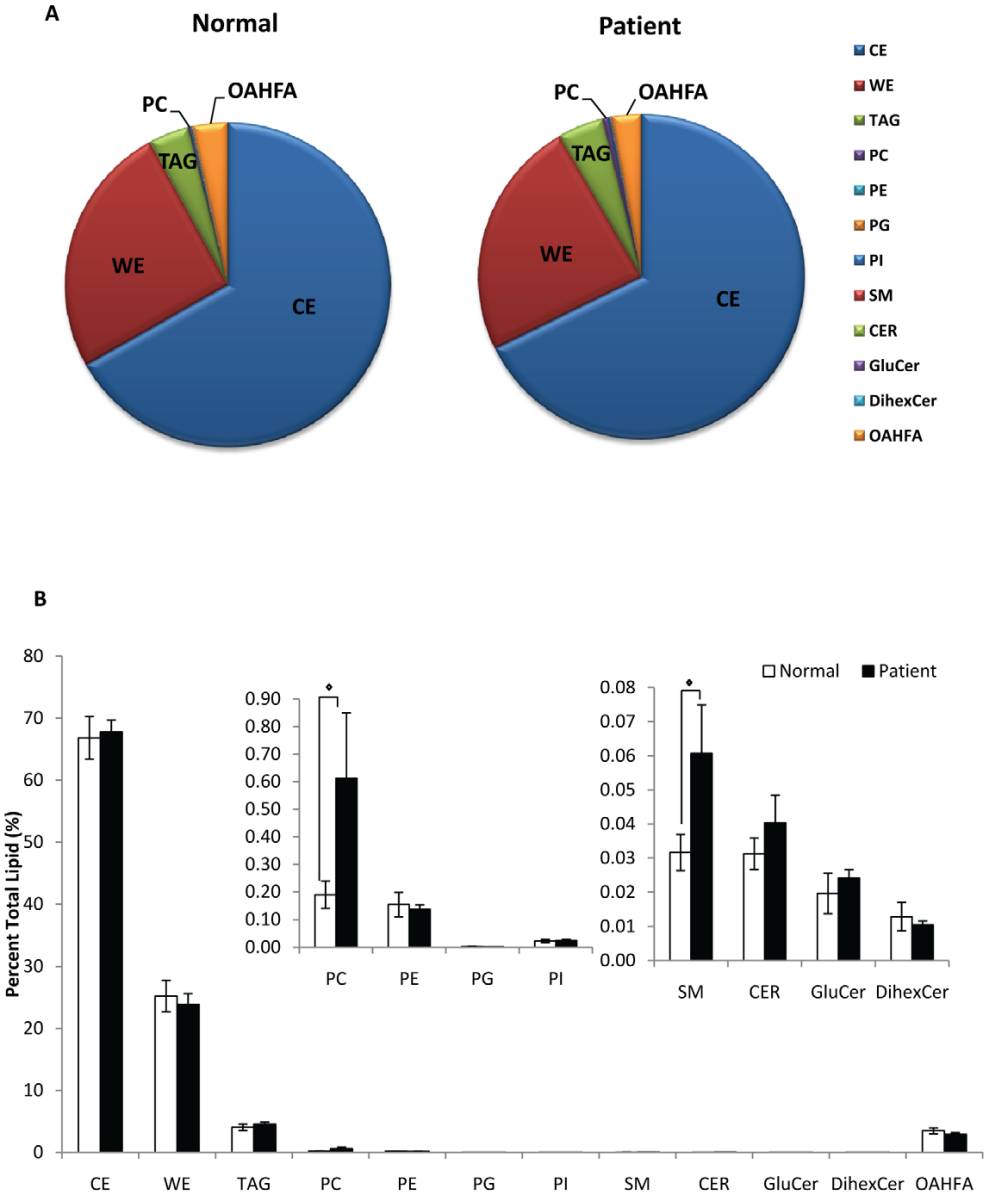
Major lipid classes in human meibum. (A) Overall distribution of major lipid classes in human meibum in normal subjects (n = 10) and DTS patients (n = 27). (B) Comparison on the quantity of major lipid classes between normal subjects (n = 10) and patients (n = 27). Insert, phospholipids (PC, PE, PG, PI) and sphingolipids (SM, Cer, GluCer, DihexCer). ◊ p<0.10, * p<0.05, ** p<0.01.

Analysis of the total lipid fraction of individual classes of lipid was shown in Figure 4. No appreciable difference was observed in the overall lipid distribution between meibum obtained from patients and normal controls (Figure 4). Similar to the trend observed in normal controls, the non-polar lipid classes of CE, WE and TAG constitute the bulk of the lipids in the meibum, with CE being present in proportionately higher levels than both WE and TAG; and the class of OAHFA forms the majority of the polar lipid fraction in both groups (Figure 4A). While both groups of individuals displayed similar general lipid profiles, detailed analysis of the polar lipid fraction revealed that the levels of PC (p = 0.093) and SM (p = 0.064) were higher in patients than in normal controls (Figure 4B, insert). Notably, the total fraction of PC in patients was approximately threefold of that in normal controls with marginal significance (Figure 4B, insert).

The levels of individual lipid species in normal controls and patients were quantified and the differences were illustrated by the heat plots in Figure S5. Notably, a number of highly unsaturated TAG species, including TAG/54:7, TAG/54:5, TAG/56:7 and TAG/56:6 were significantly higher (p<0.05) in patients than control subjects (Figure S5A). On the other hand, numerous polar lipid species displayed strikingly different profiles between the two groups. A number of PC species was found in significantly higher levels in patients compared to normal controls, and it was noted that these species either belong to the subclass of lysoPCs or contain double bond(s) within their structures (*i.e.* ether/plasmalogen PCs or unsaturated PCs), with the exception of PC32:0 (Figure S5B). A similar trend was observed for unsaturated PI species (Figure S5C). Also noteworthy is that GluCer d18:0/16:0 (p<0.01), GluCer d18:0/18:0 (p<0.01); as well as GluCer d18:0/24:0 (p<0.05) were significantly higher in the meibum of patients than that in normal controls (Figures S5D). A number of sphingolipid species, including SM18:0/16:0, Cer d18:1/16:0 and Cer d18:1/22:0, were significantly increased (p<0.05) in the meibum of patients compared to that in controls (Figures S4A–B).

The TBUT of the patients was significantly reduced compared to the controls (p<0.05) (Table S3). The mean TBUT of the patients and controls was 2.9±1.1 s and 5.7±4.1 s, respectively.

## Discussion

### Lipidome of Human Meibum

#### CEs were found in greater abundance than WEs

In concordance with previous studies [24], [32], cholesterol represents the dominant sterol in the SE pool of the meibum (Figure 2A), therefore, subsequent analysis using selective ion monitoring (SIM) was focused on the detection and quantification of cholesterol-based ester species. The CE species detected with highest abundance included CE24:0, CE25:0 and CE26:0, which is in agreement with Chen et al. [24] and Nicholaides et al. [12]. Other abundant species included CE18:1, CE20:0, CE24:1 and CE30:1. Consistent with the observation made by Butovich et al. [21], a group of highly hydrophobic compounds that eluted after the VLC-CEs reported above, with retention time at approximately 18–19 min, was detected, which could possibly represent CE species with chain lengths greater than C-32 (Figure 2C), but their quantification was not attempted in the current study. Single-stage MS was performed to obtain the elemental composition of WE species. Our preliminary results indicated that WE species contained a variety of fatty acids, with oleic acid (C18:1) as the predominant species (data not shown), which is consistent with previous reports [15], [23]. The possible contribution by WE species with other fatty acid moieties, such as (C16:1)-based WEs that were earlier reported by Chen et al. [24], could not be excluded although their quantification was not attempted in the current study. In concordance with Butovich et al. [23], the major fatty alcohols (FAl) found in WEs were of a saturated nature and ranged from C17 to C30, although considerable amounts of their monounsaturated counterparts were also present (Figure S1B).

Contrary to earlier reported studies [13], [14], [24], the most abundant class of non-polar lipids detected was CEs instead of WEs, which comprises more than half (approximately 67%) of the total lipids in the human meibum (Table 1). This stark difference in the levels of CEs may be due to differences in the methods of analysis used in estimating the quantity of individual lipid species in the meibum, such as alterations in the approaches employed for separation and ionization of the individual lipid classes present in the meibum. Moreover, in the current study, a single standard (cholesteryl stearate-26,26,26,27,27,27(d6) and palmityl palmitate) was used for quantification of the entire class of CE and WE species, respectively. This could also lead to errors in the quantitative estimation of individual CE, and especially WE species in this study. Most WE species detected in meibum are oleic acid (C18:1)-based and unsaturated in nature. To our knowledge, there is no commercially available standard suitable, such as deuterated or ^13^C-labelled WE species, for use in the accurate quantification of meibum WE species. While palmityl palmitate (C16:0C16:0) is not present endogenously in the human meibum, it is considerably shorter than most endogenous WE species. Due to its shorter chain length and thus faster elution in LC than other WE species, the quantification of WEs in the current study might be influenced by the matrix effects. In order to achieve absolute quantification, isotopically labelled standards with structures identical to each individual CE or WE species would be needed to minimize the effect of ion suppression on the quantification of individual lipid species. Ongoing experiments are being conducted to address this issue.

The quantity of CEs detected in the study group (27 patients and 10 normal controls) ranged from 38.7% to 82.6% (Figure S6A), while that for WEs ranged from 11.1% to 49.5% (Figure S6B). This indicated that the altered ratio of CEs to WEs observed between different studies could be partly due to minor variations in sampling techniques, e.g. the force applied onto eyelids to express the meibum. Other factors, such as differences in the ethnicities of the study cohorts, could also possibly account for the observed differences in the overall distribution of CE between different studies.

#### Highly unsaturated TAG species were detected

A notable finding in the current study was the detection and quantification of several highly unsaturated TAG species (n≥4) with relatively low abundance, which had not been previously reported. This indicates a high degree of unsaturation in the fatty acid moieties that constitute such TAG species, implying their susceptibility to lipid peroxidation. Such TAG species might therefore be of potential relevance to inflammatory processes associated with DTS pathogenesis [33] despite their low abundance in the human meibum.

#### Both phospholipids and OAHFAs are possible candidates for the amphiphilic layer

The occurrence of phospholipids in human meibum has been a matter of contention, as numerous phospholipid species that had been reported earlier [11], [13], [16] using chromatography were not robustly detected in later studies [19], [24] targeted at elucidating the meibum lipidome. Earlier report had estimated that the amount of phospholipids typically present made up less than 0.05% of the total meibum lipids [8], while total phospholipids in this current study was estimated to constitute approximately 0.40% of total lipids.

Phospholipid species present in the meibum, such as PC34:2 and PC34:1, were consistently detected in all samples tested, which is in contrast to earlier report stating that phospholipids were not detected at all in many of the samples tested [8]. The definitive identification of phospholipids in the current study is supported by the recent findings from Saville et al. [34], in which the presence of several phospholipid species belonging to the classes of SM and PC in both the human meibum and tears were reported. The consistent detection of phospholipids in all samples tested in the current study, despite in relatively low abundance, suggests that this class of lipids might be inherently present in the meibum. Moreover, since the meibomian glands are holocrine glands and the individual acinar cells are excreted *in toto* [6], it is thus not surprising that phospholipids originating from the cell membranes constitute a natural source of polar lipids in the meibum. Nevertheless, it cannot be ruled out that the controversial presence of phospholipids in meibum samples could have been due to differences in sampling techniques. For instance, varying amounts of force applied onto the eyelids to express the secretion from the glands would have resulted in different amounts of cell debris in the meibum collected. Further investigation is needed to confirm the presence and origin of phospholipids in the human meibum.

The presence and origin of phospholipids in the human meibum have remained intriguing topics; because phospholipids had been proposed as possible candidates for the amphiphilic layer of TFLL [6], [13], [16]. The amphiphilic component of the TFLL is critical for maintaining the structural integrity of the tear film as a whole, and has been proposed as a crucial intermediate player in ensuring the even spreading of the non-polar lipid layer together with the aqueous layer during each blink of the eye [6]. While current findings suggest that phospholipids remain likely candidates for the amphiphilic layer, it is also important to consider the physiological roles of OAHFAs, a relatively new class of amphiphilic compounds first confirmed in the human meibum by Butovich et al. [23]. It was stated that on a structural basis, the estimated ratio of 1 amphiphilic molecule per 20 nonpolar ones is essential for maintaining the bulk of nonpolar molecules in TFLL [8]. In this study, the amount of OAHFAs in the meibum was estimated to be approximately 3.5% of the total meibomian lipids, which seemed to fit the required ratio relatively well. It is also possible that species of phospholipids and OAHFAs, as well as other polar lipids detected, such as the sphingolipids, function synergistically as the amphiphilic layer to maintain the structural stability of TFLL.

### Changes in Lipid Profiles in Relation to Dry Eye Disease Progression and Disease Onset

In order to circumvent the limitation imposed by the age difference between DTS patients and control subjects, patients of similar ages were categorized into three severity levels based on OSDI (Table S1). Furthermore, it is of substantial clinical importance to distinguish between different levels of DTS severity (*i.e.* mild, moderate and severe) for effective diagnostic and prognostic purposes on a clinical basis [35]. Comparison in the changes of lipid profiles between DTS patients and normal controls were also reported in the current study in an attempt to reveal lipid species that might be pathologically relevant to DTS onset. Nevertheless, further validation using age-matched controls and larger sample size is essential to verify the current results reported.

Similar to dry eye disease, increasing severity of meibomian gland dysfunction (MGD) was reported to be associated with worse symptoms (OSDI) [36]. It was previously shown that increasing MGD severity in dry eye patients from Asia were associated with increased levels of certain tear proteins [37]. In the current study, however, the differences in lipid levels between severity groups were not explained by differences in the severity of MGD based on clinical signs, in view of the similar TBUT and clinical features of meibomian glands. Nevertheless, without performing more extensive investigation such as meibography, we cannot be certain of the extent of glandular loss in these patients.

We found lower TBUT in the patient group compared to the controls, suggesting that tear stability in dry eye patients is reduced. This validated the classification system we adopted from Sullivan et al. [29] based on dry eye symptom thresholds. Early MGD is very common in Asian populations, and control participants had level 1 and level 2 MGD [36]. The patient group was found to have level 2 and level 3 MGD, which may explain the greater tear film instability. Since MGD is a major cause of evaporative dry eye, the fact that more severe MGD is correlated to more severe dry eye or DTS is not surprising.

While conventionally perceived as major components of cellular membranes and lipid droplets with largely structural roles, lipids have now emerged as key players serving distinct biochemical functions in a wide range of biological processes such as signalling events, trafficking, and compartmentalization of macromolecules [38]. Similarly, lipid species that are critically involved in DTS pathogenesis can either serve structural roles in maintaining the structural integrity of TFLL or function as mediators in various biochemical pathways implicated in the disease, or both. Inflammation and oxidative stress are two primary events that underlie the a number of disease pathologies, and are often associated with lipid peroxidation that leads to the formation of various bioactive lipids, including oxidized phospholipids (OxPLs), short chain reactive aldehydes, platelet activating factor (PAF), oxidized CEs, oxidized free fatty acids, lysophospholipids (LPCs) and oxysterols [39].

In the current study, we had identified a number of inflammation-associated bioactive lipid species, such as various LPCs, as well as their unsaturated phospholipid precursors that significantly differed in quantity amongst patients of different severity levels, as well as between patients and control subjects. This finding was corroborated by a previous comprehensive study on protein biomarkers in tear fluid, which had identified a correlation between dry eye severity and the levels of proteins associated with inflammatory response, including α1-acid glycoprotein 1, S100A8 and S100A9 [35].

#### Unsaturated species of TAGs and phospholipids can possibly exert both biochemical and structural effects in dry eye disease pathogenesis

A relatively consistent trend was observed for a number of saturated and unsaturated TAG species, which were significantly decreased in the moderate category compared to the mild category. In an earlier model of TFLL proposed by McCulley and Shine, TAGs and WEs were suggested as transitional lipids that facilitate the bridging between the polar and non-polar lipid phases [13]. In their proposed model, it was stated that TAG unsaturation is critical for maintaining the stability of the polar lipid phase as well as in promoting the proper segregation of non-polar and polar lipids in the meibum, as interactions between TAG and PC depend on the degree of fatty acid unsaturation within TAG at a temperature range of 35°C–37°C [40]. As such, the drop in the levels of unsaturated TAG species in moderate category of patients compared to the mild patients could possibly have an adverse effect on TFLL stability. Biochemically, the unsaturated fatty moieties inherent in highly unsaturated TAG species, which were found to be significantly upregulated in DTS patients compared to controls, can possibly predispose patients to ocular inflammatory attacks associated with DTS onset due to the higher susceptibility of such species to lipid peroxidation.

Increasing knowledge on the role of phospholipase A2 (PLA_2_) in the maintenance of ocular homeostasis and pathology of ocular-related diseases has become available in the recent decade [41]. For instance, increased PLA_2_ activity was found in patients with chronic blepharitis compared to normal controls [42]. PLA_2_s had been previously detected in both the conjunctiva and corneal epithelial tissues adjacent to the tear film, and specifically cleave the acyl ester bond at the sn-2 position of phospholipids to yield free fatty acids and lysophospholipids [41]. The hydrolysis of phospholipids can result in the release of free fatty acids and lysophospholipids, both of which can potentially function as lipid signalling molecules that can exert an array of physiological effects including inflammatory reactions [43], contributing to the pathogenesis of DTS. Thus, we speculate that the observed increase in the levels of lysophospholipids found in patients could possibly be attributed to increased PLA_2_ activity and the subsequent hydrolysis of phospholipids. Phospholipid-bound polyunsaturated fatty acids (PUFAs) are also principal targets for events of oxidative attacks [44]. Therefore, the increased levels of unsaturated and plasmalogen/ether PCs in patients would theoretically predispose the affected individuals to the disease by increasing the chances of OxPL formation via oxidative attacks, leading to a series of pro-inflammatory responses that essentially underlie DTS pathogenesis.

While a number of lysophospholipids and ether/plasmalogen phospholipids were found in increased levels in patients compared to controls, the opposite trend was observed for patients in the severe category. These lipid species aforementioned were decreased in the severe category when compared to the moderate category with marginal significance. Culminating evidence now suggest that OxPLs can exert both pro- and anti-inflammatory effects depending on the biological context [45]. Thus, changes in the levels of OxPLs and their precursor phospholipids in the meibum of DTS patients might be either due to inflammatory assaults associated with disease progression, or a result of defensive mechanisms of the meibomian gland cells or corneal epithelial cells to elicit anti-inflammatory responses in an attempt to maintain cellular homeostasis.

Structurally, it had been suggested that the presence of unsaturated fatty acids in phospholipids results in demixing or segregation of the polar lipid layer, which could potentially destabilize the tear film as a consequence [13]. In-depth analysis into the precise roles of individual OxPLs as well as their quantification in DTS patients versus control subjects is needed to construct a more holistic view of the lipid-mediated inflammatory responses associated with DTS. The complex interplay of inflammatory-associated and structural functions exhibited by the various phospholipid species might possibly explain their fluctuating levels as disease severity increases.

#### OAHFAs might represent suitable indicators of dry eye disease progression

In contrast to phospholipids, OAHFA species displayed a consistently decreasing trend as disease severity increases. This supports their putative role in maintaining the structural integrity of TFLL by serving as candidates for the amphiphilic layer to facilitate interactions between the polar and non-polar lipids. Their clear and discernible trend across different disease severity levels might possibly allow them to function as indicators of disease progression. Verification of the observed trends in OAHFA species with larger sample size is needed before a conclusive statement can be made.

#### Patterns of lipid changes suggested the multifactorial nature of dry eye disease pathogenesis

It is also noteworthy that while non-polar lipid species such as TAGs and CEs were found to be significantly different between mild and moderate categories of patients, it is the polar lipid profiles that were markedly different between the moderate and severe categories. Possibly, changes in non-polar lipid layer might be implicated in the early phase of DTS, probably via disturbing the TFLL integrity and thus increasing the rate of evaporation and depletion of the aqueous layer underneath. McCulley and Shine had earlier suggested that the aliphatic hydrocarbon chain lengths of non-polar lipids such as CEs, WEs and TAGs determine the cohesiveness and water vapour transmission rate of the non-polar lipid layer [13]. At a later stage of the disease, however, alterations in polar lipid profiles, especially those associated with inflammation might be responsible for the various clinical manifestations that are commonly observed in the more severe cases of DTS, which are predominantly caused by conditions of hyperosmolarity attributed to the loss of an intact TFLL to retard evaporation in early-phase DTS.

#### Sphingolipids might be critical in maintaining the structural integrity of TFLL

It was notable that the class of SM lipids was appreciably increased in the meibum of patients with marginal significance (p = 0.064). McCulley and Shine had suggested that sphingolipids, especially cerebrosides, are crucial players in initiating the segregation of polar lipids from the more non-polar lipids in the presence of water molecules, and a decreased amount of cerebrosides was found in the meibum of patients with chronic blepharitis [13]. While an opposite trend was observed in this current study, it must be noted that overall structural stability of the tear film should depend on a delicate homeostatic balance of lipid species, especially those that carry out fundamental roles in stabilizing the TFLL. Therefore, sphingolipids can be potentially important in promoting the structural integrity of TFLL. Also of interest was the observation that a number of GluCer species including GluCer d18:0/16:0, GluCer d18:0/18:0 and GluCer d18:0/24:0 were significantly increased in patients. Increased levels of Cer had been observed in patients with meibomian keratoconjunctivitis (MKC) due to abnormal hyperkeratinization of the meibomian gland ducts resulting in their obstruction [14]. Liu et al. had also recently confirmed that keratinization plays a critical role in the pathogenesis of MGD via genome-wide analysis of lid tissues obtained from normal controls and MGD patients [46]. In a recent study, it was found that increasing Cer to meibum ratios *in vitro* had an appreciable impact on the stability of human meibum films, suggesting that higher than optimal level of Cer decreases the stability and elasticity of the TFLL leading to its collapse [47], supporting the structural roles elicited by Cer species in TFLL.

#### Limitations of the current study design

There was a lack of age-matched control subjects for unbiased comparison of lipid profiles with that of DTS patients (Tables S2, S3), who were of a significantly older age than the control subjects recruited due to higher incidence of DTS with increasing age. It had been previously reported that aging exerts a significant effect on the lipid profiles of a number of polar and neutral lipid species in the human meibum [17]. Thus, certain changes observed in the current study could possibly be confounded by the effect of age. However, as only m/z values but not the specific identities of the lipid species had been reported [17], we were unable to do a comparison with results obtained in this study.

Gender was also not perfectly matched in the current study, although a larger proportion of female subjects were found in both patient and control groups (Table S2). Also, it was more difficult to recruit male DTS patients due to female individuals having a greater predisposition to the disease.

Moreover, the multifactorial nature and complicated manifestations of ocular surface disease make it almost impossible to rely solely on any single biomarker for disease diagnosis and monitoring. This implies that currently there is no single diagnostic test that can be used as a proxy for severity level of dry eye or MGD for evaluating lipid levels. On the other hand, a panel of tear biomarkers is probably required to rationally classify dry eye and MGD for the purpose of treatment and clinical trials.

In conclusion, the current study represents the first attempt to provide an insight into sieving out the pathologically relevant lipid species for dry eye disease on scale of the entire lipidome, which can undoubtedly facilitate a more comprehensive understanding of the disease itself. In essence, more drastic quantitative differences were observed in minor lipid species of lower abundance compared to nonpolar species (CEs and WEs) that constitute the bulk of the meibum amongst patients of different severity levels, as well as between DTS patients and normal controls. Amongst the various lipid classes investigated, OAHFAs represent the only class of lipids with consistently decreasing levels that correlate with increasing disease severity, which renders these lipids suitable indicators of dry eye disease progression. Nevertheless, it is crucial to recognise that age is a confounding factor in the current experimental design that cannot be overlooked, and ongoing work is being conducted to recruit age-matched controls for verification of the results reported.

## Materials and Methods

### Chemicals

Chloroform and methanol were purchased from Merck (Merck Pte. Ltd., Singapore). Ammonium hydroxide (28% in H_2_O) and palmityl palmitate were purchased from Sigma-Aldrich (St. Louis, MO, USA). Deionized water was obtained from a MilliQ purification system (Millipore, Bedford, MA, USA). DMPC, DMPE, DMPG, DMPS, C17-Cer, C8-GluCer and C12-SM were obtained from Avanti Polar Lipids (Alabaster, AL, USA). Dioctanoyl phosphatidylinositol PI-8:0/8:0 was purchased from Echelon (Echelon Biosciences, Inc., Salt Lake City, UT, USA). OAHFA 18:1/16:0 was synthesized as previously described [23] and used as an internal standard for quantitation of OAHFA species. Cholesteryl stearate-26,26,26,27,27,27(d6) and TAG48:0(d5) were purchased from CDN Isotopes Inc. (Quebec, Canada).

### Study group

In total, 42 patients and 13 control subjects were recruited for this study (Figure 1). The 42 patients were diagnosed with dry eye syndrome at Singapore National Eye Center by Dr Louis Tong. The patient samples were further reduced to 27 (24 females and 3 males; average age 59.4, range 28–75) by including only patients with OSDI>21 (Table S2). Control subjects (7 females and 3 males; average age 29.4; range 22–45) were defined as healthy volunteers with OSDI≤12.9. Informed consent was obtained from all participating subjects and the procedure for the project was specifically approved by the SingHealth Centralised Institutional Review Board (CIRB Reference No. 2008/611/A). Written consent was obtained from all participants involved in the study. Clinical examinations included subjective symptoms, Schirmer's test type I (without anaesthesia), tear breakup time (TBUT) and corneal fluorescein staining. For TBUT, a drop of normal saline was instilled on the fluorescein strip (Fluorets) then shaken off so that no visible drop remained. The subject was asked to look up before the introduction of the fluoret to the inferior conjunctival fornix on the right then left eye. The participant was then asked to blink a few times and close the eyes for few seconds without blinking. He/She was then asked to open the eyes, look ahead at the observer's forehead and not blink for as long as possible. TBUT was defined as the time between the lid opening and the first appearance of any dry spot on the cornea. The subject was then requested to close his eyes for few seconds and the procedure repeated for left eye. The Schirmer's test was done with the standard strips currently used (5 mm wide with a notch for folding). No prior anaesthetic was used before the test and the strips were positioned over the inferior temporal half of the lower lid margin in both eyes at the same time. Corneal fluorescein staining was evaluated in each subject after TBUT determination, since the instillation of fluorescein had been done. The documentation of staining spots was performed under slit lamp biomicroscopy and documented in the 5 zones of the cornea (central, superior, inferior, nasal and temporal) as reported by Barr et al. in the CLEK study [48]. Clinical microscopic features of MGD such as loss of expressibility of meibomian glands, alteration in the viscosity of the meibum expressed, irregularity of meibomian gland orifices, and eyelid notching or telangiectasia were recorded, if present.

### Meibum collection and sample preparation

Human meibum was collected by gently squeezing the eyelids of subjects and the meibum released immediately solidified at room temperature. The expressed meibum was collected using a metal spatula. Meibum lipids collected were eluted into an eppendorf tube by washing the metal spatula thoroughly with 900 µL of chloroform: methanol (1∶1). The lipid extracts were dried using speed-valco (Thermo Savant, milford, USA) and stored at −80°C until further analysis. The dried meibum samples were reconstituted in chloroform: methanol (1∶1) prior to analysis by high-performance liquid chromatography coupled with mass spectrometry (HPLC/MS).

### Quantitative analysis of lipids using HPLC/MS

Lipid profiles were analyzed using the 3200 and 4000 Q-trap® LC/MS/MS systems. Neutral lipids were analyzed using a sensitive HPLC/ESI/MS method [49]. Briefly, separation of triglycerides (TAGs) from polar lipids was carried out on an Agilent Zorbax Eclipse XDB-C18 column (i.d. 4.6×150 mm). Selective ion monitoring (SIM) was used to record CE, WE and TAG species. TAGs were calculated as relative contents to the spiked d5-TAG 48:0 internal standard, while CEs and WEs were normalized to cholesteryl stearate-26,26,26,27,27,27(d6) and palmityl palmitate spiked into the samples, respectively. An Agilent high performance liquid chromatography (HPLC) system coupled with an Applied Biosystem Triple Quadrupole/Ion Trap mass spectrometer (4000Qtrap) was used for quantification of individual polar lipids (phospholipids, sphingolipids and OAHFAs). Based on product ion and precursor ion analysis of head groups, multiple reaction monitoring (MRM) transitions were set up for quantitative analysis of various polar lipids [50]. Levels of individual lipids were quantified using spiked internal standards including dimyristoyl phosphatidylcholine PC (28:0-PC), dimyristoyl phosphatidylethanolamine (28:0-PE), dimyristoyl C14-phosphatidylserine (28:0-PS), dimyristoyl phosphatidyglycerol (28:0-PG), dimyristoyl phosphatidic acid (28:0-PA), and dioctanoyl phosphatidylinositol (PI, 16:0-PI). N-lauroyl-D-erythro-sphingosylphosphorylcholine (C12-SM), N-heptadecanoyl-D-erythro-sphingosine (C17-Cer), and D-glucosyl-ß-1,1′ N-octanoyl-D-erythro-sphingosine (C8-GluCer) were used for the quantification of different sphingolipid classes. OAHFA 18∶1/16∶0 was used as an internal standard for quantification of OAHFA species.

The relative level of each individual lipid was normalized using the following equation:
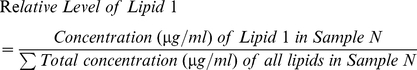
The individual percentage for each class of lipids in a particular sample was calculated as follows:
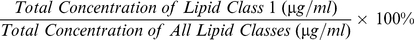



### Study Design and Statistical Analysis

Quantitative comparisons were made between 27 patients and 10 control subjects for all 256 lipid species analysed using the Student's t test to identify lipid species that were significantly different between patients and control subjects (p<0.05 was considered to be statistically significant and 0.05≤p<0.10 was considered as marginally significant) (Figure 1). In order to account for age as a confounding factor in the analysis of lipid profiles between patients and control subjects, patients were stratified into three groups (*i.e.* mild, moderate and severe) based on OSDI score which offers a measure of symptom severity (Table S1). There was no significant age differences between the 3 groups by ANOVA (p>0.05). The lipid levels in patients from the three categories were compared using one-way ANOVA with post hoc TukeyHSD test (p<0.05 was considered to be statistically significant and 0.05≤p<0.10 was considered as marginally significant) to correlate changes in lipid levels with DTS progression.

## Supporting Information

Table S1 Demographic data and OSDI score of DTS patients in the mild (n = 12), moderate (n = 12) and severe (n = 11) categories. Tear breakup time, TBUT; Schirmer's test 1, Schirmer's; OSDI, ocular surface disease index.
(TIF)

Table S2 Demographic data and OSDI score of DTS patients (n = 27) and normal subjects (n = 10). Tear breakup time, TBUT; Schirmer's test 1, Schirmer's.
(TIF)

Table S3 Summary of the age and clinical indicators (Schirmer's, TBUT, OSDI scores) of DTS patients (n = 27), normal subjects (n = 10), patients from the mild (n = 12), moderate (n = 12) and severe (n = 11) categories. Tear breakup time, TBUT; Schirmer's test 1, Schirmer's; OSDI, ocular surface disease index. Values were presented as means ± standard errors. ^¥^Mild: OSDI≤21.0; ^€^Moderate: 21.0<OSDI≤41.2; ^£^Severe: OSDI>41.2. OSDI cutoffs for mild, moderate and severe categories of patients were modified from Sullivan et al. [29].
(TIF)

Figure S1 **Distribution of non-polar lipid species in human meibum for normal subjects (n = 10) and patients (n = 27).** (A) CE, cholesteryl esters; (B) WE, wax esters; (C) Triacylglycerides, TAG. ◊ p<0.10, * p<0.05, **p<0.01.
(TIF)

Figure S2 **Distribution of (O-acyl)-ω-hydroxy-fatty acid (OAHFA) species in human meibum for normal subjects (n = 10) and patients (n = 27).** **◊** p<0.10, * p<0.05, **p<0.01.
(TIF)

Figure S3 **Distribution of phospholipid species in human meibum for normal subjects (n = 10) and patients (n = 27).** (A) PC, phosphatidylcholines; Insert, PC species that significantly differed between normal subjects and patients; (B) PE, phosphatidylethanolamines; (C) PI, phosphatidylinositols; (D) PG, phosphatidylglycerols. ◊ p<0.10, * p<0.05, **p<0.01.
(TIF)

Figure S4 **Distribution of sphingolipid species in human meibum for normal subjects (n = 10) and patients (n = 27).** (A) SM, sphingomyelins; (B) Cer, ceramides; (C) GluCer, glucosylceramides; (D) dihexCer, dihexosylceramides. ◊ p<0.10, * p<0.05, **p<0.01.
(TIF)

Figure S5 **Heatplots of individual species from various lipid classes that were significantly different between normal subjects (n = 10) and patients (n = 27).** (A) TAG, Triacylglycerides; (B) PC, Phosphatidylcholines; (C) PI, Phosphatidylinositols; (D) GluCer, Glucosylceramides. ◊ p<0.10,* p<0.05, ** p<0.01. * Higher in patients; * lower in patients.
(TIF)

Figure S6 **Scatter plots illustrating the individual percentage of (A) CE, cholesteryl esters; and (B) WE, wax esters; found in the meibum for all 37 subjects within the study group.**
(TIF)
