# Supplementary figures and images for: Meibum Lipid Composition in Asians with Dry Eye Disease
Source: PLoS One. 2011 Oct 17;6(10):e24339. doi: 10.1371/journal.pone.0024339 (PMC3197196; doi:10.1371/journal.pone.0024339)

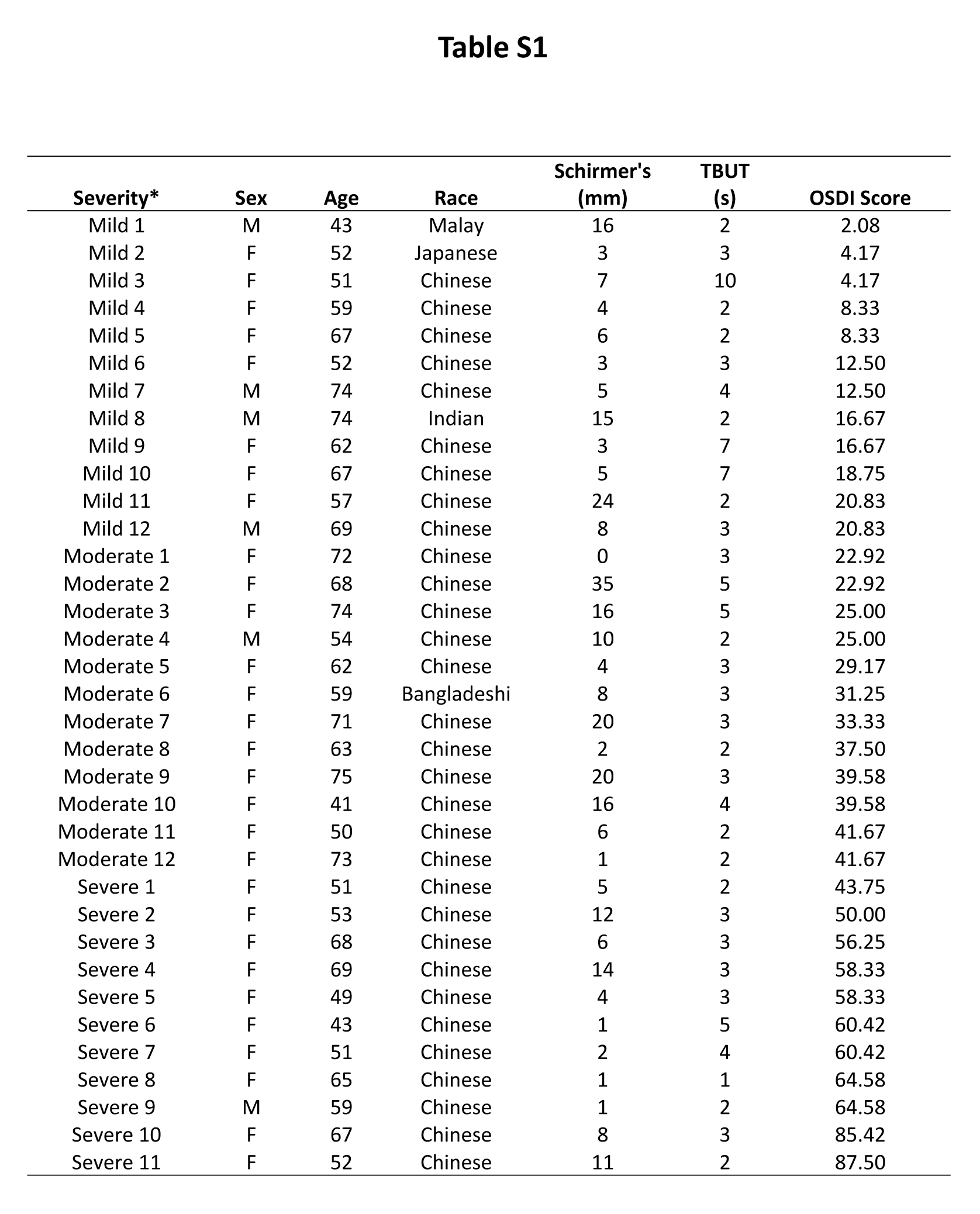

Supplement: Table S1 — Demographic data and OSDI score of DTS patients in the mild (n = 12), moderate (n = 12) and severe (n = 11) categories. Tear breakup time, TBUT; Schirmer's test 1, Schirmer's; OSDI, ocular surface disease index. (TIF) [file pone.0024339.s001.tif]

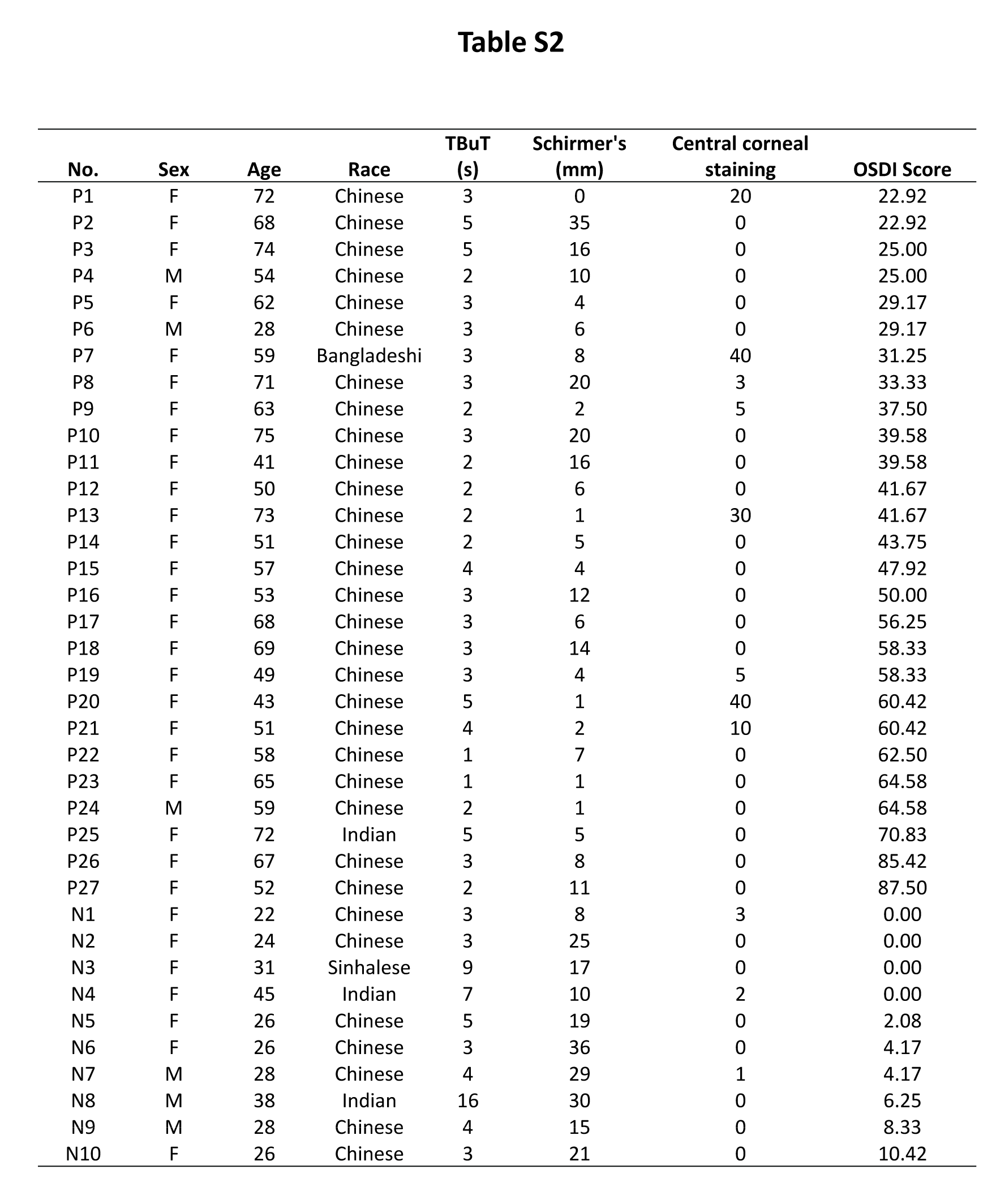

Supplement: Table S2 — Demographic data and OSDI score of DTS patients (n = 27) and normal subjects (n = 10). Tear breakup time, TBUT; Schirmer's test 1, Schirmer's. (TIF) [file pone.0024339.s002.tif]

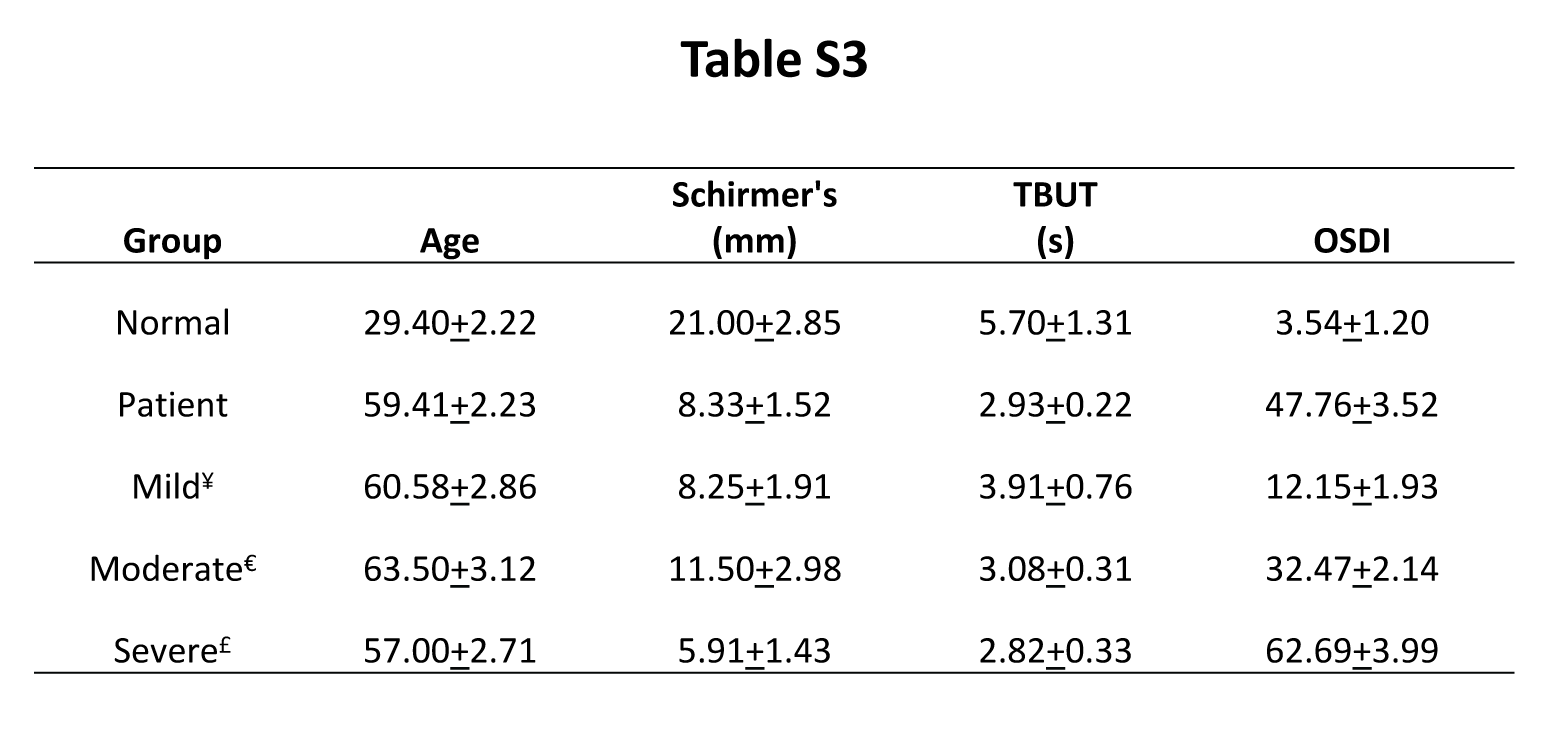

Supplement: Table S3 — Summary of the age and clinical indicators (Schirmer's, TBUT, OSDI scores) of DTS patients (n = 27), normal subjects (n = 10), patients from the mild (n = 12), moderate (n = 12) and severe (n = 11) categories. Tear breakup time, TBUT; Schirmer's test 1, Schirmer's; OSDI, ocular surface disease index. Values were presented as means ± standard errors. ¥Mild: OSDI≤21.0; €Moderate: 21.0<OSDI≤41.2; £Severe: OSDI>41.2. OSDI cutoffs for mild, moderate and severe categories of patients were modified from Sullivan et al. [29]. (TIF) [file pone.0024339.s003.tif]

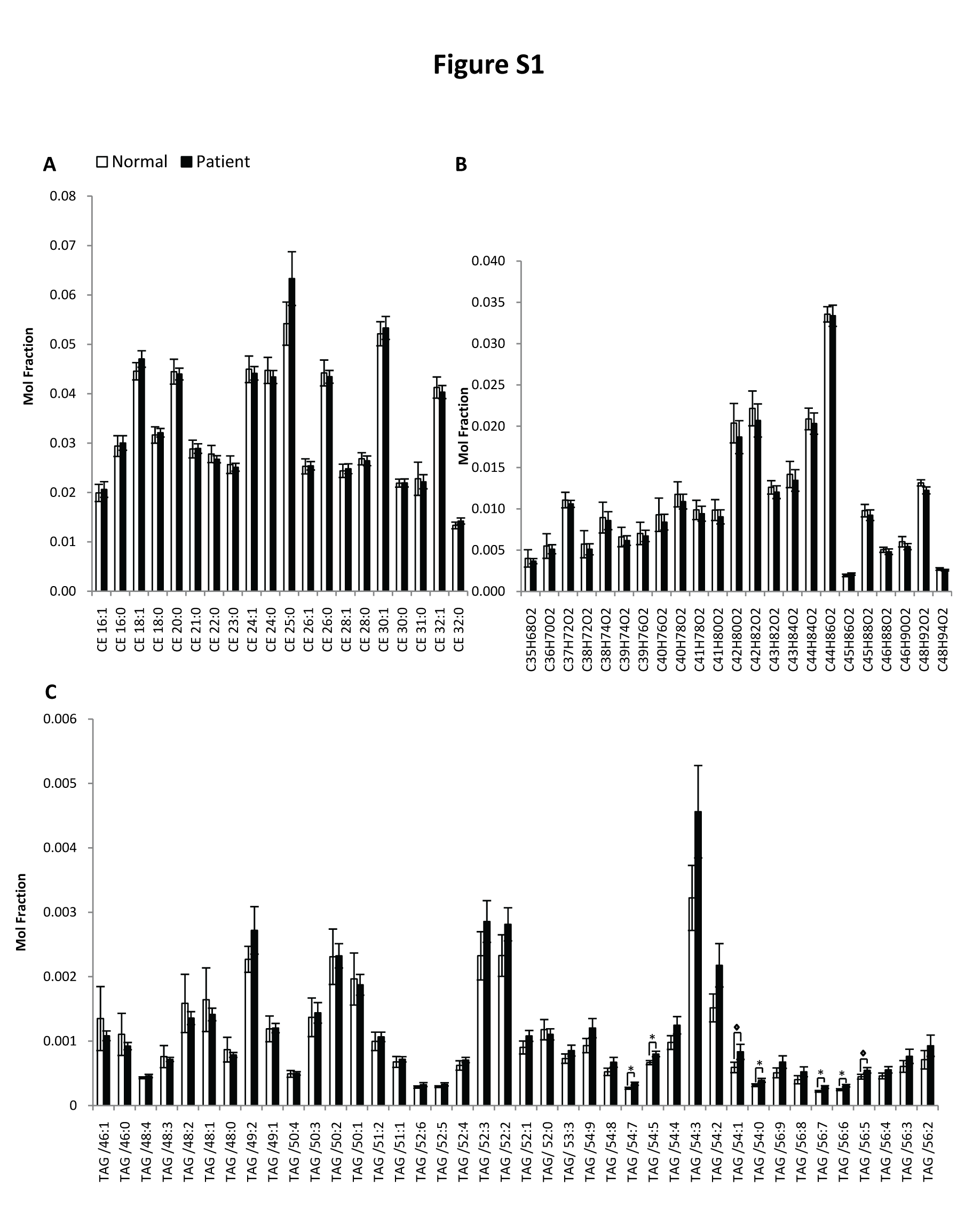

Supplement: Figure S1 — Distribution of non-polar lipid species in human meibum for normal subjects (n = 10) and patients (n = 27). (A) CE, cholesteryl esters; (B) WE, wax esters; (C) Triacylglycerides, TAG. ◊ p<0.10, * p<0.05, **p<0.01. (TIF) [file pone.0024339.s004.tif]

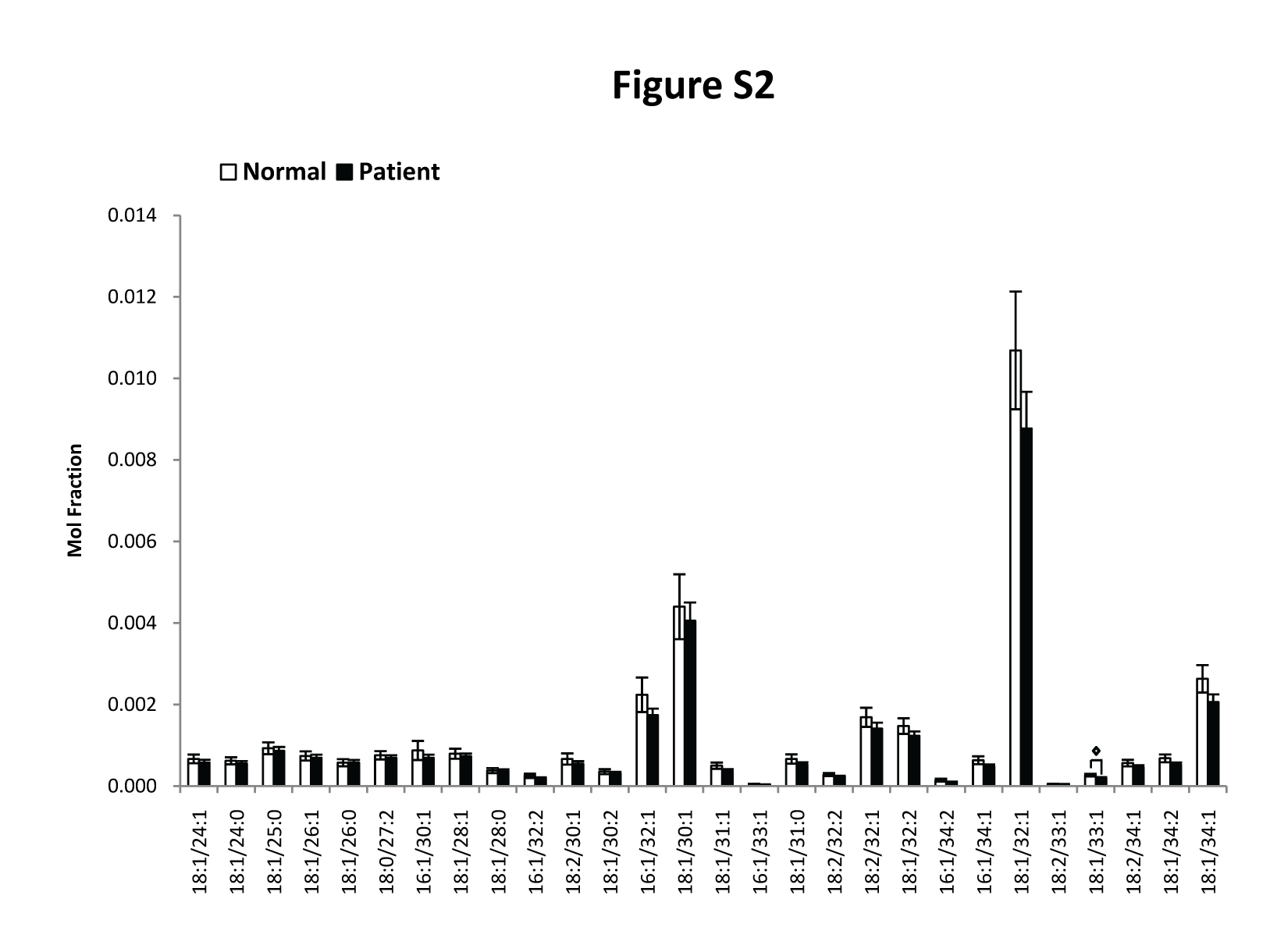

Supplement: Figure S2 — Distribution of (O-acyl)-ω-hydroxy-fatty acid (OAHFA) species in human meibum for normal subjects (n = 10) and patients (n = 27). ◊ p<0.10, * p<0.05, **p<0.01. (TIF) [file pone.0024339.s005.tif]

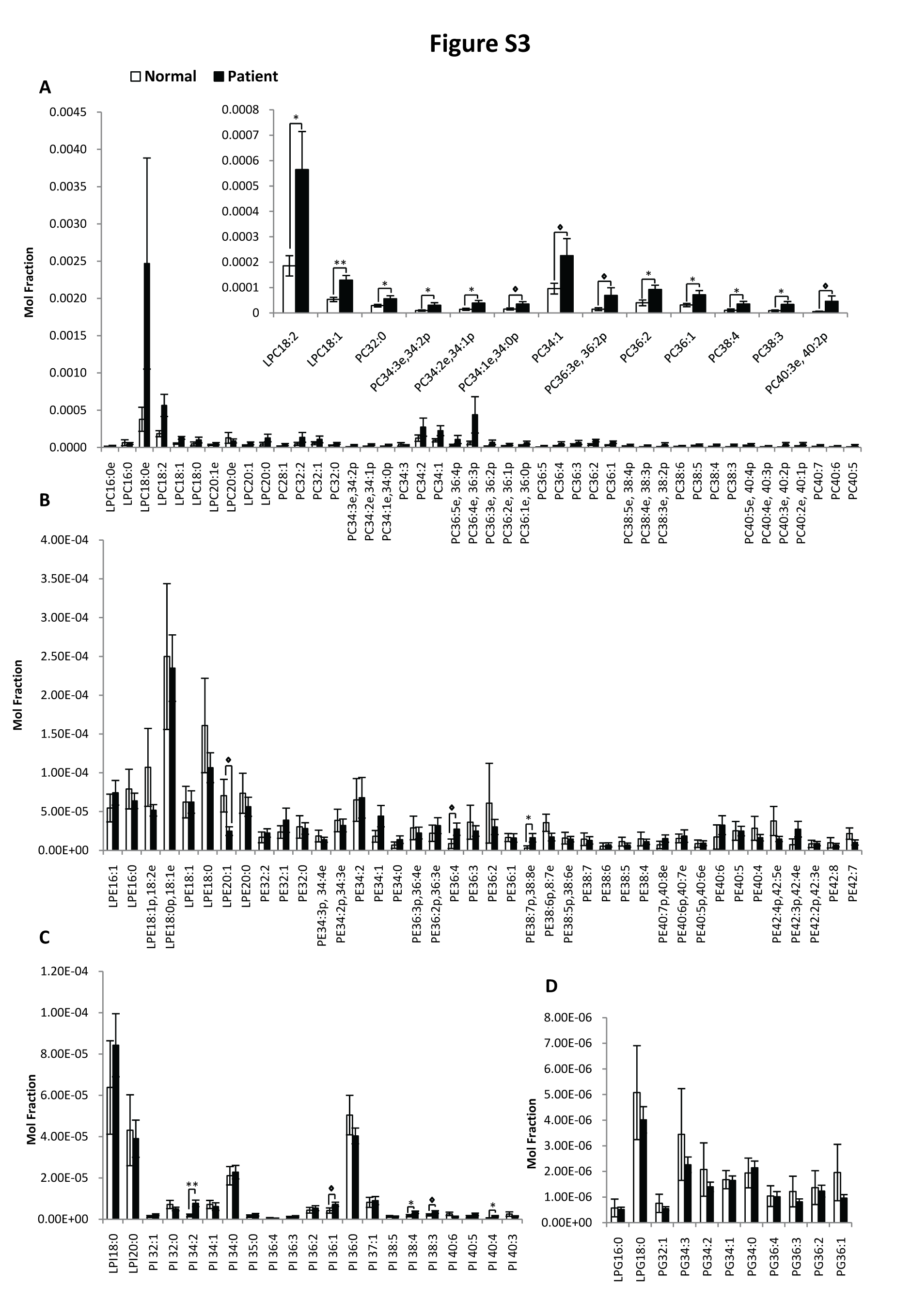

Supplement: Figure S3 — Distribution of phospholipid species in human meibum for normal subjects (n = 10) and patients (n = 27). (A) PC, phosphatidylcholines; Insert, PC species that significantly differed between normal subjects and patients; (B) PE, phosphatidylethanolamines; (C) PI, phosphatidylinositols; (D) PG, phosphatidylglycerols. ◊ p<0.10, * p<0.05, **p<0.01. (TIF) [file pone.0024339.s006.tif]

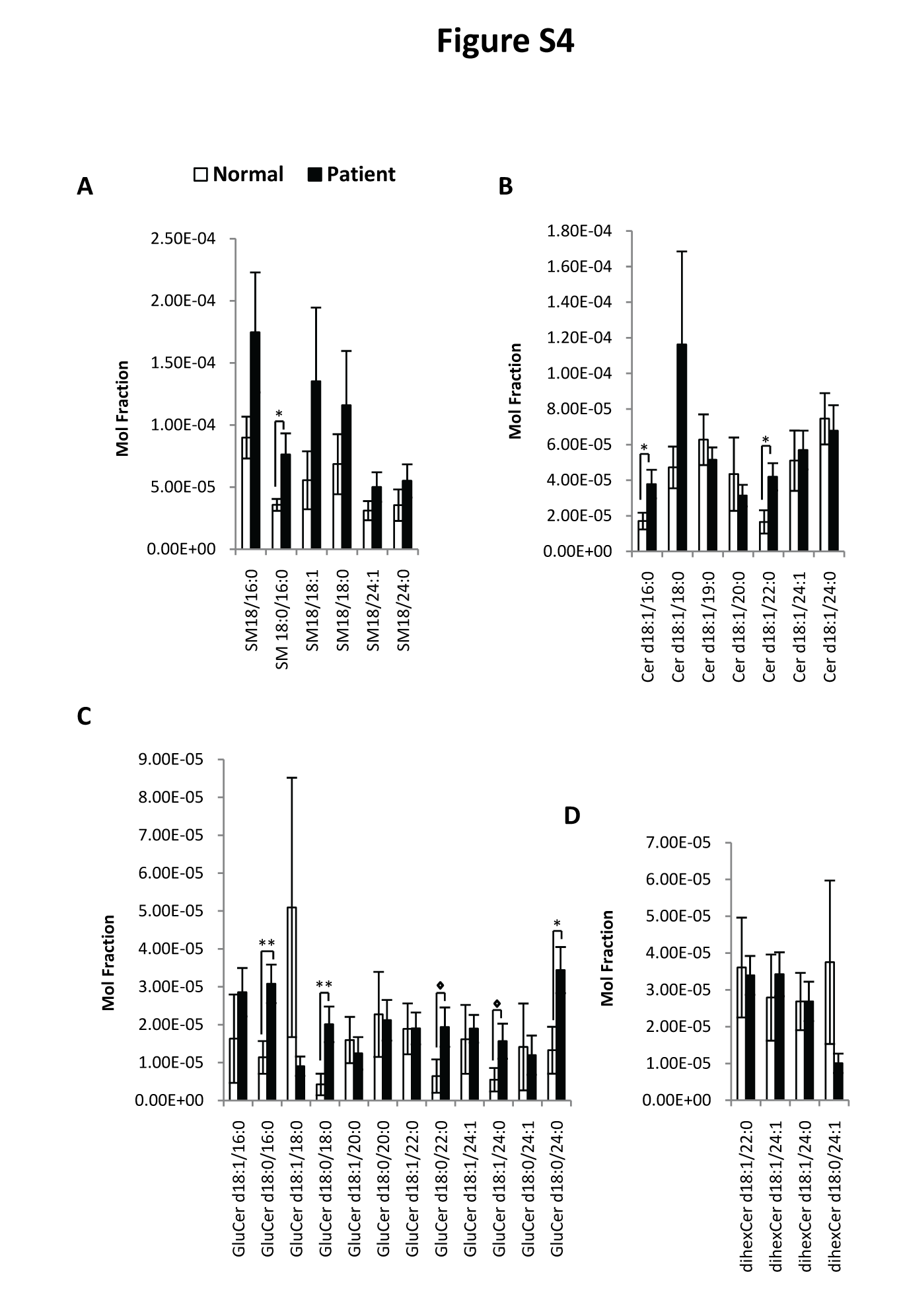

Supplement: Figure S4 — Distribution of sphingolipid species in human meibum for normal subjects (n = 10) and patients (n = 27). (A) SM, sphingomyelins; (B) Cer, ceramides; (C) GluCer, glucosylceramides; (D) dihexCer, dihexosylceramides. ◊ p<0.10, * p<0.05, **p<0.01. (TIF) [file pone.0024339.s007.tif]

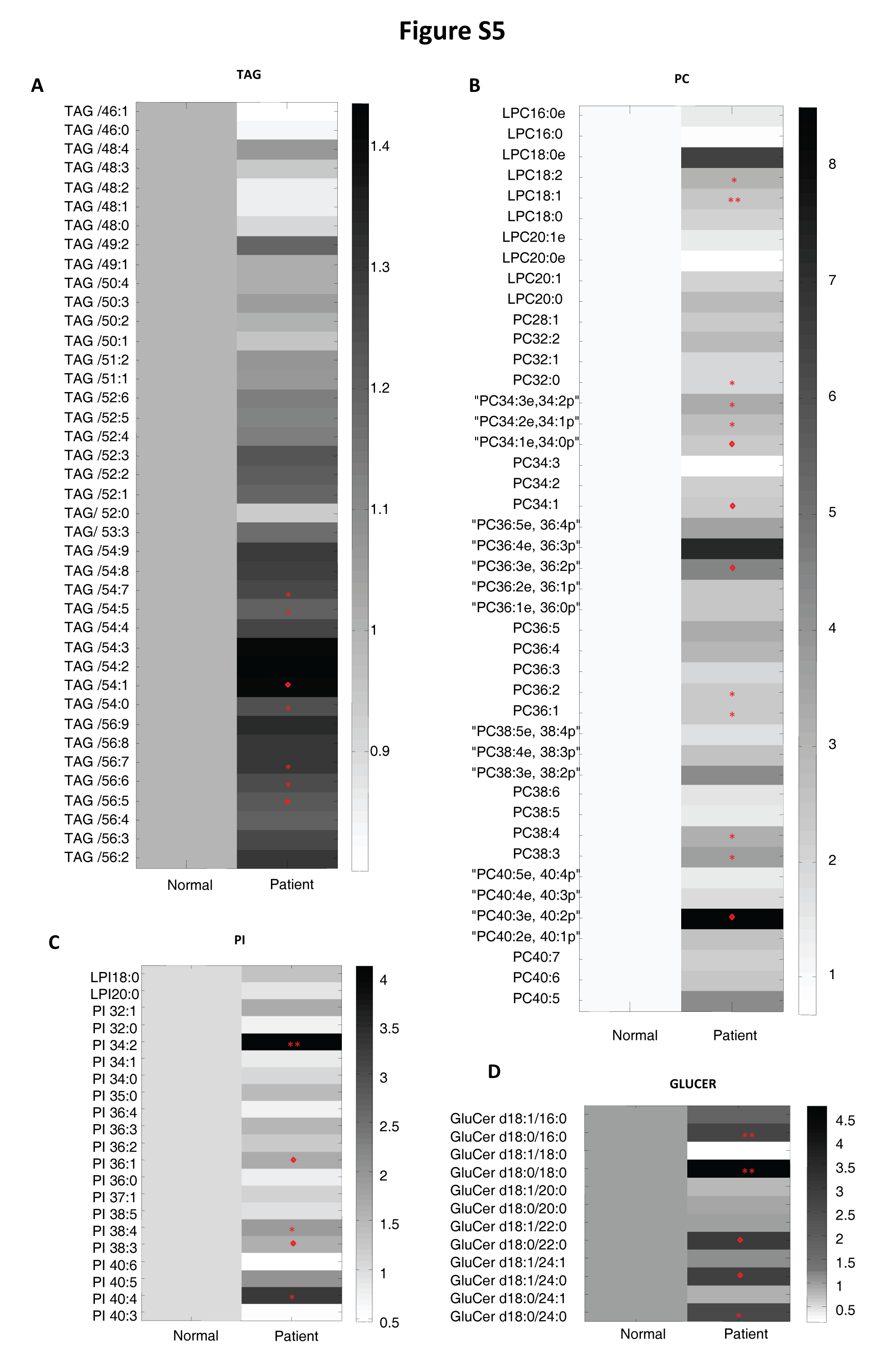

Supplement: Figure S5 — Heatplots of individual species from various lipid classes that were significantly different between normal subjects (n = 10) and patients (n = 27). (A) TAG, Triacylglycerides; (B) PC, Phosphatidylcholines; (C) PI, Phosphatidylinositols; (D) GluCer, Glucosylceramides. ◊ p<0.10,* p<0.05, ** p<0.01. * Higher in patients; * lower in patients. (TIF) [file pone.0024339.s008.tif]

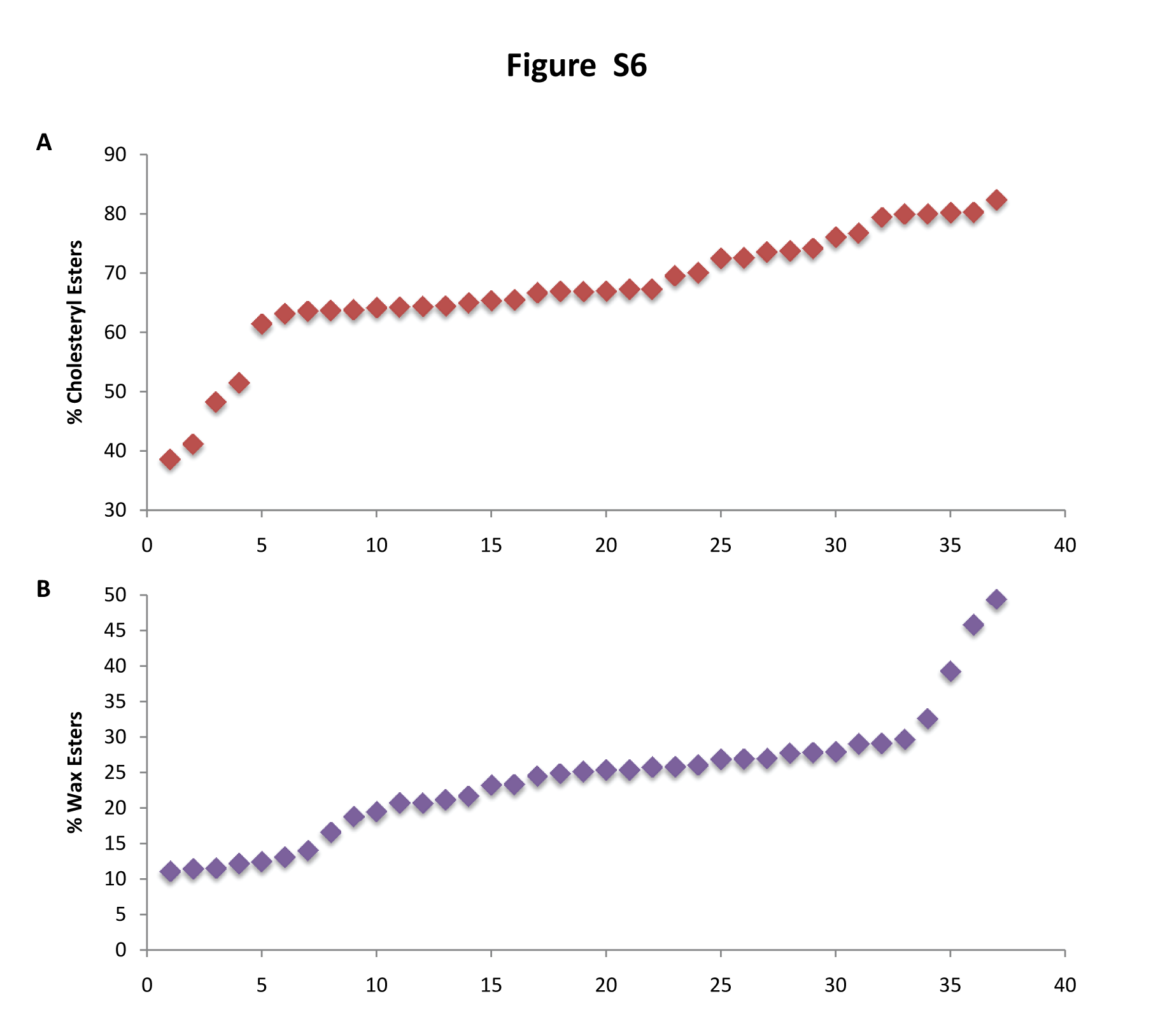

Supplement: Figure S6 — Scatter plots illustrating the individual percentage of (A) CE, cholesteryl esters; and (B) WE, wax esters; found in the meibum for all 37 subjects within the study group. (TIF) [file pone.0024339.s009.tif]
